# Microarray analysis of human keratinocytes from different anatomic sites reveals site-specific immune signaling and responses to human papillomavirus type 16 transfection

**DOI:** 10.1186/s10020-018-0022-9

**Published:** 2018-05-16

**Authors:** Mohd Israr, David Rosenthal, Lidia Frejo-Navarro, James DeVoti, Craig Meyers, Vincent R. Bonagura

**Affiliations:** 1 The Feinstein Institute for Medical Research, Manhasset, NY, USA; Division of Allergy and Immunology, Department of Pediatrics, Donald and Barbara Zucker School of Medicine at Hofstra/Northwell, Great Neck, NY USA; 2 Department of Genomic Medicine, Otology and Neurotology Group CTS495, Centre for Genomics and Oncological Research, Pfizer/Universidad de Granada/Junta de Andalucía (GENYO), Granada, Spain; 3 Department of Microbiology and Immunology, The Pennsylvania State University College of Medicine, Hershey, PA USA

**Keywords:** Immune responses, Immune pathways, HPV, Keratinocytes, Microarrays

## Abstract

**Background:**

Stratified human keratinocytes (SHKs) are an essential part of mucosal innate immune response that modulates adaptive immunity to microbes encountered in the environment. The importance of these SHKs in mucosal integrity and development has been well characterized, however their regulatory immunologic role at different mucosal sites, has not. In this study we compared the immune gene expression of SHKs from five different anatomical sites before and after HPV16 transfection using microarray analyses.

**Methods:**

Individual pools of human keratinocytes from foreskin, cervix, vagina, gingiva, and tonsils (HFKs, HCKs, HVKs, HGKs and HTLKs) were prepared. Organotypic (raft) cultures were established for both normal and HPV16 immortalized HFKs, HCKs, HVKs, HGKs and HTLKs lines which stably maintained episomal HPV16 DNA. Microarray analysis was carried out using the HumanHT-12 V4 gene chip (Illumina). Immune gene expression profiles were obtained by global gene chip (GeneSifter) and Ingenuity pathway analysis (IPA) for each individual site, with or without HPV16 transfection.

**Results:**

We examined site specific innate immune response gene expression in SHKs from all five different anatomical sites before and after HPV16 transfection. We observed marked differences in SHK immune gene repertoires within and between mucosal tracts before HPV 16 infection. In addition, we observed additional changes in SHKs immune gene repertoire patterns when these SHKs were productively transfected with HPV16. Some immune response genes were similarly expressed by SHKs from different sites. However, there was also variable expression of non-immune response genes, such as keratin genes, by the different SHKs.

**Conclusions:**

Our results suggest that keratinocytes from different anatomical sites are likely hard wired in their innate immune responses, and that these immune responses are unique depending on the anatomical site from which the SHKs were derived. These observations may help explain why select HPV types predominate at different mucosal sites, cause persistent infection at these sites, and on occasion, lead to HPV induced malignant and benign tumor development.

**Electronic supplementary material:**

The online version of this article (10.1186/s10020-018-0022-9) contains supplementary material, which is available to authorized users.

## Background

Stratified human keratinocytes (SHKs) are important immunologic components of both healthy and diseased mucosal surfaces in addition to their established role as physical epithelial barriers to infection. Accumulating evidence shows that SHKs from various mucosal tracts are important in mucosal development, inflammation, and HPV-induced cancer development (Wu et al. 2011; Saenz et al. 2008; Swamy et al. 2010; Nestle et al. 2009; Strid et al. 2009). Responses of SHKs can cause immune dysregulation (Swamy et al. 2010; Nestle et al. 2009; Strid et al. 2009; Albanesi et al. 2005; Tonel and Conrad 2009), however they also support the maintenance of the mucosal microbiome, via defensin expression, and they preserve mucosal homeostasis (Chung and Dale 2004; Frohm et al. 1997). Taken together, the importance of SHKs in both innate and adaptive immunity at mucosal sites is compelling (Swamy et al. 2010). Unresolved is the mechanism(s) that render these cells resistant or permissive to select viruses within a given viral family, such as human papillomaviruses (HPVs).

Towards understanding how oral cavity-derived keratinocytes influence adaptive immunity, Wu et al. (Wu et al. 2011) performed a global gene expression analysis that showed the significant effect of murine oral keratinocytes on adaptive immunity (Wu et al. 2011).

We previously reported that human oral tissues are permissive to HPV16 infection and that HPV replication can spread via the oral cavity (Israr et al. 2016). Here we compared the immune gene expression of SHKs using microarray analyses of pooled human keratinocytes from tonsil, foreskin, uterine cervix, vagina, and gingiva, before and after HPV16 transfection. We also compared immune gene network expression by SHKs taken from each of these anatomical sites to determine how they respond to HPV16 transfection. We observed marked differences in SHK immune gene expression within a given mucosal tract and by those derived from different mucosal tracts. In addition, we observed additional changes in SHK immune gene expression patterns when these SHKs were productively transfected with HPV16.

## Methods

### SHKs cultures, generation of HPV16 positive cell lines

Individual pools of primary SHKs from foreskin, cervix, vagina, gingiva, and tonsils (HFKs, HCKs, HVKs, HGKs and HTLKs) were grown as previously reported (McLaughlin-Drubin et al. 2004; McLaughlin-Drubin and Meyers 2005). Organotypic (raft) cultures were established as described (Meyers et al. 1997; McLaughlin-Drubin et al. 2005). Each pool of SHKs was prepared from 3 to 5 individual healthy donors. Both normal and HPV16 immortalized HFKs, HCKs, HVKs, HGKs and HTLKs lines which stably maintained episomal HPV16 DNA, and were seeded onto rat tail type 1 collagen matrices with J2 3 T3 feeder cells (Israr et al. 2016).

HPV16 infection of these SHKs could be more closely analogous to a natural infection than transfection. However, HPV16 infection of keratinocytes results in low efficiency and high variability between cell lines. Second, cells often lose more virus during further propagation (i.e.) in raft cultures, while HPV16 transfection provides high uptake efficiency, high cell line consistency, and rare heterogeneity. In addition, during transfection cells stably maintain the HPV genome as an episome, which is a critical step for a productive HPV16 life cycle and persistent HPV infection. Furthermore, integration of the HPV16 genome into keratinocyte DNA suppresses virus expression, and prevents formation of small, circular HPV16 genomes that can be packaged and transmitted to a new host keratinocyte (McBride and Warburton 2017).

### RNA isolation and microarrays analysis

Triplicate cultures, of each SHK pool were harvested, and total RNA isolated using an RNeasy mini kit (Qiagen). Microarray analysis was carried out using the HumanHT-12 V4 gene chip (Illumina) containing 47231 probes targeting > 25,000 human genes. Expression data (baseline + HPV infected for each site) was log transformed and normalized to the median. Initial **c**omparisons were performed using GeneSifter software and a volcano plot constructed (*p* = 0.05). Genes with ≥ 2 fold expression changes were considered significant in these analyses.

### Comparison of gene expression by SHKs from different mucosae

A list of “immunologic genes” was obtained by querying http://ctdbase.org/ using key words “immu*, interleukin, cytokine, and defensin”. Duplicates were removed, resulting in 2576 unique genes. A search for these genes in GeneSifter was done, sorted by expression using Kruskal-Wallis, (p cutoff = 0.02) resulting in 81 differentially expressed genes. A principle component analysis (PCA) was performed (dots = genes; lines with arrows = tissue types; axis 3 principle components) and the tissue types were separated using PC1, PC2, and PC3, to show differential gene expression patterns for these immunologic genes.

We then performed a pairwise analysis (normalized to all medians, *p*-value cutoff< 0.05) comparing individual SHKs from a single mucosal tract before and after HPV16 transfection. Among all differentially expressed genes (DEGs), a subset of genes known to be involved in immune responses/associated with immune regulation was selected for analysis. Expression data was analyzed using Ingenuity pathway analysis software: (IPA®, QIAGEN Inc., https://www.qiagenbioinformatics.com/products/ingenuitypathway-analysis). Networks were generated using the Core analysis tool (Kramer et al. 2014).

To better visualize the immunological genes uniquely, and/or commonly expressed by SHKs from each of the different anatomical sites, with or without HPV transfection, we applied these data sets to software at http://bioinformatics.psb.ugent.be/webtools/Venn/ to generate a 5 intersection Venn/Euler diagram (Fig. 2e) showing which DEGs were in expressed at each intersection.

## Results and discussion

### Keratins and immune gene mRNA expression by SHKs with or without HPV16 transfection

First we examined site specific keratins expression in all five different anatomical sites with or without HPV16 transfection that is consistent with published reports (Chu and Weiss 2002). As anticipated, all of the SHKs expressed some of keratin genes, see Additional file 1: Figure S1. However, there was variability in the repertoire of keratin genes that were expressed by different SHKs, suggesting that the cultures of pooled primary keratinocytes grown in rafts maintains the individual gene expression profiles characteristic of the site(s) from which the cells were obtained (Chu and Weiss 2002). As expected, the majority of immune genes having significant levels of expression were expressed at similar levels by all SHK cultures studied, see Fig. 2f. Significantly all 3 GAPDH probe sets, both HPRT-1 probe sets and all 3 B-actin probe sets were detected in all samples at equal levels, as anticipated, since all expression data was normalized prior to analysis. In addition to several keratins present in all tissue types (heat map), IFNAR1 and IFNGR1 and IFNGR2 were detected at equivalent levels in all samples tested, as well as IL-10 and the IL-10RB. Also, both the IL-4R and the IL-13R were expressed by all keratinocytes studied, and the expression of these genes was unaffected by introducing HPV16.

**Fig. 1:**
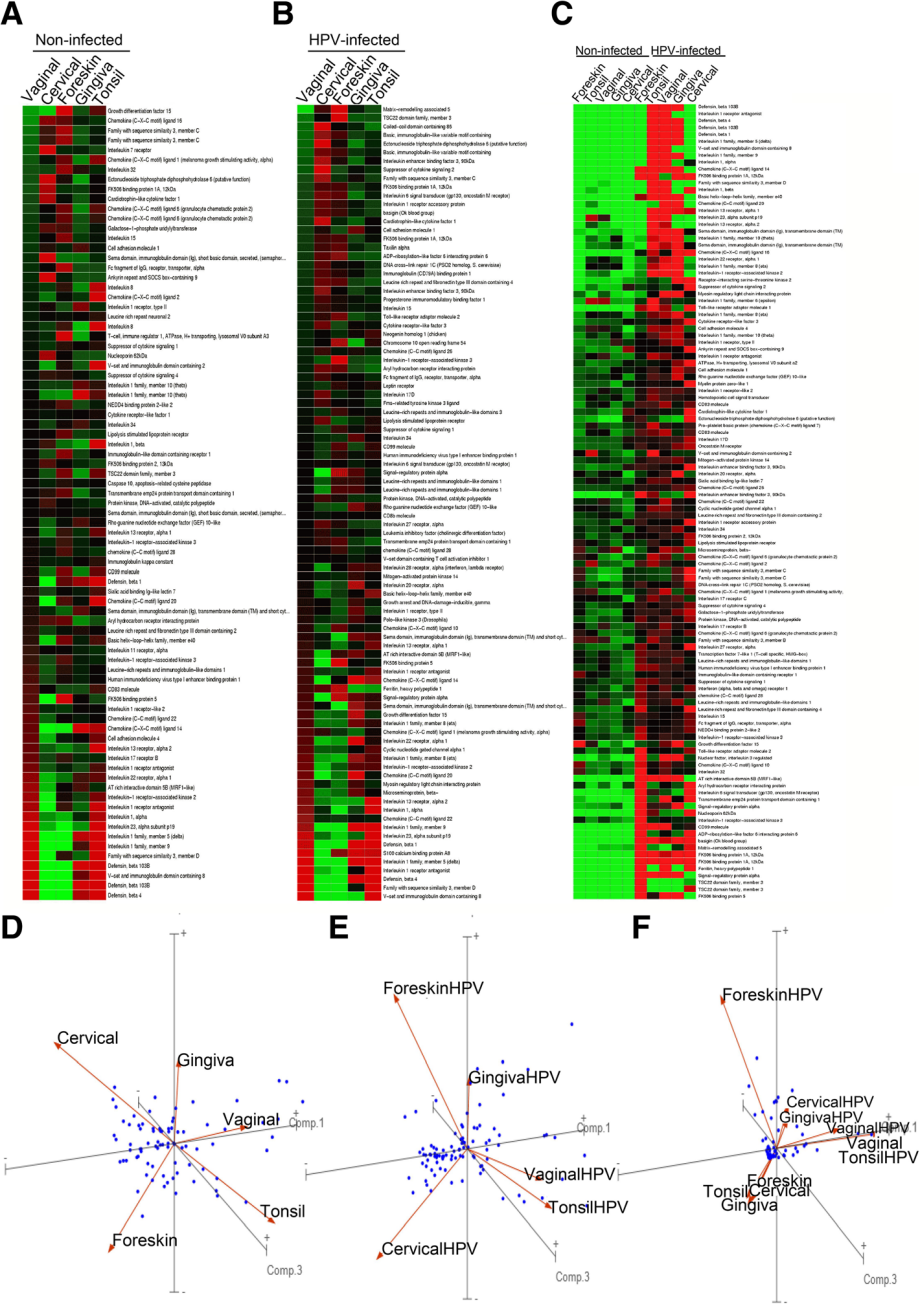
Immune gene mRNA repertoires of stratified human keratinocytes from different anatomical sites vary significantly. Results are for SHKs grown in organotypic cultures from a pool of 3–5 individuals and represent 3 independent samples of each of the mucosal sites shown (baseline and HPV infected). Data were log transformed, and normalized to the median. Eighty one “immunologic genes” are shown, and a principle component analysis (PCA) performed (dots = genes; lines with arrows = tissue types; axis 3 PCA). Tissue types were separated (PC1, PC2, and PC3), to demonstrate differential genetic expression patterns for these genes. Heat maps and the PCA for SHKs from each site are shown for resting (**a**, **d**), HPV16 transfected (**b**, **e**), and HPV16^−^ or HPV16^+^ keratinocytes from all sites separately grouped together (**c**, **f**)

Of note, expression of immune response genes varied by site of SHK origin, see heat map and principal component analysis (Fig. 1a, b). Cluster analysis grouped gingival, vaginal and tonsil cells together, with the latter two being more closely related. Foreskin and cervical cells grouped together, but clearly showed many differences. Within the upper digestive mucosal tract, gingival and tonsil cells also showed differences, for example mRNA expression for individual members of multiple classes of immune genes (CCL-20, CXCL2 and CXCL6, interleukins IL-1, IL-8, and IL1F9 (IL-36γ)), and cell surface receptors/adhesion molecules (IL1RII, IL-13Rα, CD99). Several defensins were expressed by SHKs from these tissues, but not by cervical or foreskin SHKs.

We examined of all of the chemokines and interleukins present on the gene microarray, and found a limited number of immunologic signaling molecules being expressed in our keratinocyte cultures, largely irrespective of the derivation of the SHKs we studied. A single CCL chemokine, specifically CCL-20 was detected in all samples, together with its receptor CCR6. 2 CXC chemokines, CXCL14 and CXCL16 were also expressed by keratinocytes as well as the CXC receptors CXCR1 and CXCR7. IL8 was also expressed by all keratinocytes studied, while IL-23a was absent from both cervical and foreskin cultures. IL1-A and IL1-B were detected in all keratinocytes studied, albeit at varying levels. However, the antagonist of both 1A and 1B, IL1RN, the IL-20 receptor, and the IL17D receptor were highly expressed by all keratinocytes studied. IL-18 was equally expressed by all keratinocytes studied. In contrast, the IL-1 family member IL1F9, now called IL-36γ, and its antagonist IL1F5 were differentially expressed by different keratinocytes obtained from different anatomical sites.

HPV16 transfected cells also showed marked differences between cell types (Fig. 1b). Under these conditions, (cut off significance = 0.02), 92 different genes were identified. Similar to uninfected cells, vaginal and tonsil cells clustered together. However, gingival cell immune gene expression was quite different, while foreskin and cervical cells clustered together, although foreskin and cervical SHKs were quite different from each other. Among the most highly expressed genes by gingiva, vagina, and tonsil cells was IL-36γ. We previously reported that this cytokine was highly expressed by laryngeal papilloma cells (HPV6/11 infected) (DeVoti et al. 2008; DeVoti et al. 2014). Interestingly, IL36γ was not elevated in HPV16^+^ foreskin and cervical cells. Thus, SHKs from different mucosae show differential immune genes expression before and after HPV transfection.

Comparing SHKs from different sites as a group before or after HPV transfection (Fig. 1c) showed that SHKs before HPV16 transfection behaved more similarly to each other than to their HPV16^+^ counterparts which also behaved more similarly to each other (Fig. 1c). Two exceptions were noted, HPV16^+^ SHKs from foreskin did not express a similar repertoire of immune response genes than HPV16^+^ cells from other anatomical sites expressed, and HPV16^−^ SHKs vaginal cells expressed similar immune gene profiles as HPV^+^ cells from other mucosae (Fig. 1c).

### Immune gene mRNA networks expressed by SHKs from different sites

**Fig. 2:**
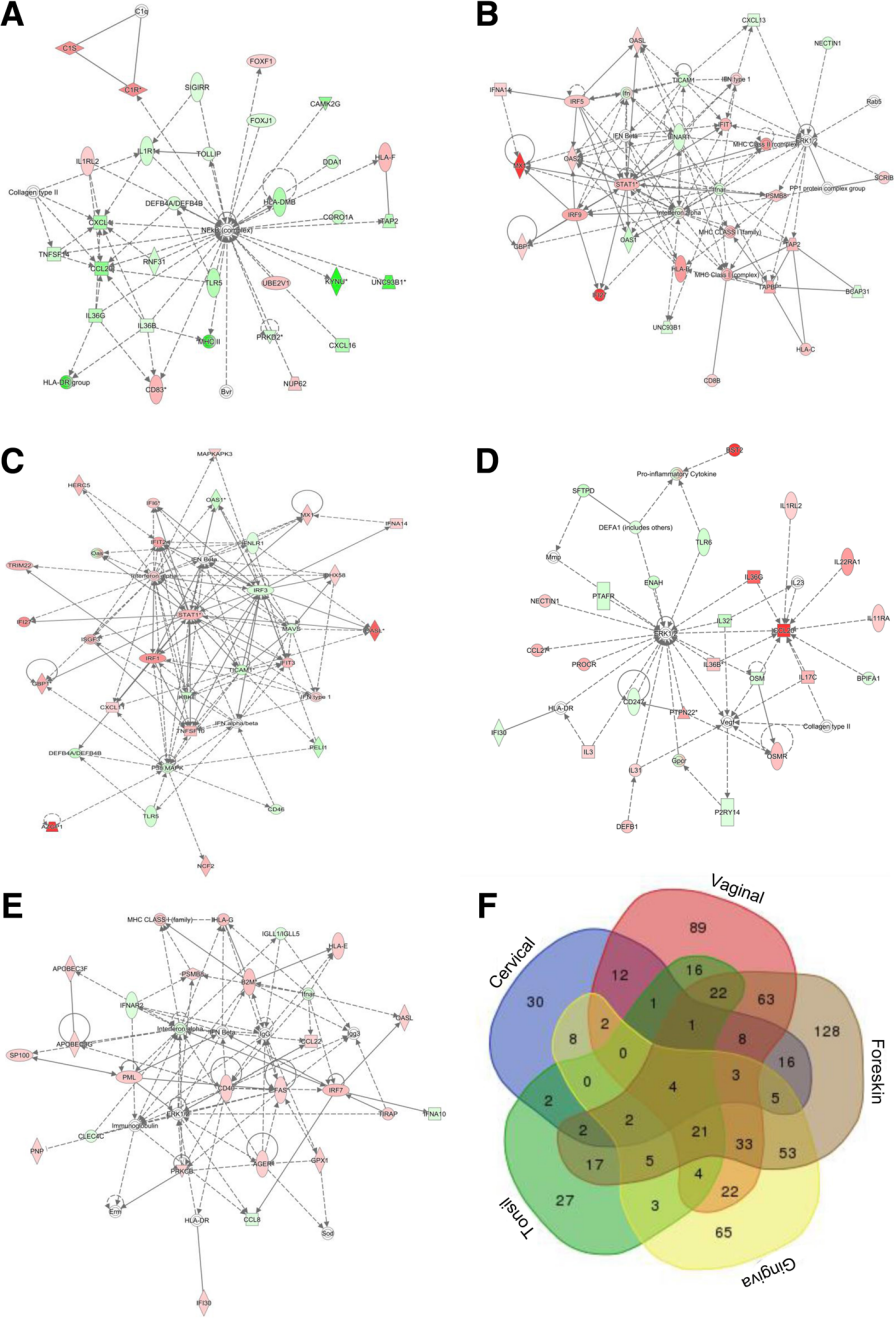
Immune gene network expression by HPV16^+^ stratified human keratinocytes from different anatomical sites vary significantly. Pairwise analysis, normalized to all medians (*p*-value cutoff < 0.05) was performed individually to comparing SHKs expression at a single mucosal site before and after HPV16 transfection. Differentially expressed genes were selected from a larger list of genes (immune responses/regulation) for the analysis. Data were analyzed using Ingenuity pathway analysis software (IPA®, QIAGEN Inc., https://www.qiagenbioinformatics.com/products/ingenuitypathway-analysis). Networks were generated using the Core analysis tool (**a**-**e**) (Kramer et al. 2014), and a Venn/Euler diagram was generated (**f**) to visualize common vs. unique gene expression by applying the data sets of SHKs (different sites, resting vs HPV16^+^ cells) to software at http://bioinformatics.psb.ugent.be/webtools/Venn/. The number of genes that were uniquely or commonly expressed by cells from each anatomical site are shown within each intersection (**f**)

We compared mRNA expression of an expanded list of immune genes and regulators by Ingenuity software, at each anatomical site with or without HPV16 transfection (Fig. 2a-e). There were significant differences in gene pathways used by SHKs in response to HPV16 transfection at each site. Differentially expressed genes in these comparisons are show in Additional file 2: Table S1. Fig. 2f (Venn/Euler diagram) shows that there were more differences in genes expressed by SHKs from each site, compared to genes expressed in common by SHKs from all sites.

In summary, immune gene expression by SHKs in health and disease has previously been studied predominantly in the oral cavity (gingiva, tonsil), the skin, and the uterine cervix (Wu et al. 2011; Chung and Dale 2004; Frohm et al. 1997; Nees et al. 2001; Adami et al. 2014). In some of these studies, mucosal biopsies from different epithelial tissues contained cells other than keratinocytes, and they showed novel cytokine expression at different anatomical sites (Frohm et al. 1997). However, there is only a single report of a genome wide analysis of oral mucosa-derived SHKs in mice that demonstrated the essential role of these cells in regulating adaptive immunity (Wu et al. 2011).

Several important points can be drawn from the results presented in this communication. The repertoire of immune response genes expressed by resting SHKs from different mucosal tract show significant differences when compared to each other (Fig. 1). These expression patterns can distinguish one mucosa from another. Even within the same mucosal tract there are marked differences in immune gene expression. These responses are likely to be “hard wired” as SHKs from different sites do not regress to express a common immune gene repertoire in organotypic culture (Fig. 1). HPV16^−^ SHKs from different anatomical sites as a group cluster together and look more similar to each other than do their HPV16+ counterparts, and vice versa. However, there were a few notable exceptions (Fig. 1c). We speculate that the mucosal immune micromilieu at different anatomical sites may influence which immune genes the keratinocytes express as it is likely the stem cells that ultimately differentiate into keratinocytes are the same for all keratinocytes at each site. We have not performed microarrays of naturally infected SHKs from these different anatomical sites, however, our in vitro transfected cervical cell lines showed similar gene expression profiles as previously published in human HPV16 cervical cancers (Nees et al. 2001; Santin et al. 2005; Perez-Plasencia et al. 2007).

Additionally, the immune gene repertoires of HPV16^+^ SHKs from different sites also differ from each other based on the origin of the SHKs (Fig. 1b). Thus, immune gene expression by these cells appears to also be based on mucosal SHK origin (Fig. 1b). Finally, immune gene networks associated with HPV16^+^ SHKs from the different anatomical sites, compared to their HPV16 non-transfected counterparts, was not uniform and significantly varied based on the anatomical origin of origin (Fig. 2a-f). The significant variation in immune gene repertoires and networks (Fig. 2f) may have bearing on why individual members of the large HPV family of DNA viruses predominate as the cause of persistent infection and disease within and between different mucosal tracts (Cubie 2013).

## Conclusions

In this study, we identify the differences and similarities in mRNA gene expression made by stratified keratinocytes obtained from five different anatomical sites, grown in organotypic cultures before and after HPV16 transduction. Our results show that keratinocytes within and outside of a given mucosal tract show different immune gene repertoires that are site specific, and that introducing HPV16 into these cells also alters their immune gene expression in a site specific manner. Thus, keratinocytes from different anatomical sites are likely hard wired in their innate immune responses, before and after the introduction of HPV16, and that these immune responses are unique depending on the anatomical site from which they were derived.

The potential medical significance of understanding and manipulating differential immunologic gene expression by keratinocytes from different mucosal anatomical sites is intriguing. It is known that at some mucosal sites, HPV 16 predominates as a potential pathogen and causes persistent infection leading to malignancy, while others, like HPV6 and 11 rarely if ever cause disease at that mucosal site. For example, tonsil and base of tongue, HPV-induced disease is caused by HPV16, but not HPV6 or 11. In contrast, HPV 6 and 11 commonly cause persist infection and “benign” respiratory papilloma development in the larynx and upper airway, while HPV16 rarely does at this anatomical site. It is interesting to speculate that differential immune gene expression by keratinocytes from a given mucosal site may convey resistance vs. susceptibility to different HPVs dependent on the repertoire of innate immune responses genes they express at a given mucosal site. This may be so given that the keratinocyte HPV receptor appears to be the same for all HPVs (Schafer et al. 2015; Raff et al. 2013). Thus, an explanation of why certain keratinocytes from a given mucosal site are resistant to a some HPVs, but susceptible to others, and vice versa for keratinocytes at different mucosal sites, may be related to the kind of innate immune signaling that a given keratinocyte expresses at a given mucosal site, before and after HPV infection. If this were the case, understanding how keratinocytes confer resistance or susceptibility to a given HPV at a given mucosal site could potentially open a novel preventative and/or therapeutic modality to block a specific HPV from causing persistent infection, and ultimately benign or malignant tumor development at a specificceptible anatomical site. These observations may help explain why select HPV types predominate at different mucosal sites, and can cause persistent infection, that on occasion, can lead to HPV induced malignancy.

## Additional files

Additional file 1: **Figure S1.** Heat map showing the relative gene expression level of keratins among five different stratified human keratinocytes with or without HPV16 transfection. To generate a keratins heat map. All keratin genes, KRT-1 to KRT-86 on the illumina human HT-12v4 expression array were aligned and assigned colors corresponding to the relative expression levels, comparing triplicate cultures of (Strid et al. 2009) types of normal human keratinocytes (NHK) with triplicate cultures from each type transfected with HPV16. KRT-12, − 18, − 20, − 22, KRT-25 to KRT-77, KRT-79, and KRT-82 to KRT-86 were excluded because all 10 triplicates had average expression levels that were below the 95th percentile of all genes on the chip. (XLSX 13 kb)

Additional file 2: **Table S1.** Genes that are differentially expressed by stratified human keratinocytes from different anatomical sites after HPV16 transfection. (DOCX 16 kb)

## Abbreviations

DEGs: Differentially expressed genes
HCKs: Human cervical keratinocytes
HFKs: Human foreskin keratinocytes
HGKs: Human gingival keratinocytes
HPVs: Human papillomaviruses
HTLKs: Human tonsil keratinocytes
HVKs: Human vaginal keratinocytes
IPA: Ingenuity pathway analysis
PCA: Principle component analysis
SHKs: Stratified human keratinocytes
