# Supplementary material for: Microarray analysis of human keratinocytes from different anatomic sites reveals site-specific immune signaling and responses to human papillomavirus type 16 transfection
Source: Mol Med. 2018 May 16;24:23. doi: 10.1186/s10020-018-0022-9 (PMC6016891; doi:10.1186/s10020-018-0022-9)
Supplement: Supplementary file 2 — Table S1. Genes that are differentially expressed by stratified human keratinocytes from different anatomical sites after HPV16 transfection. (DOCX 16 kb) [file 10020_2018_22_MOESM2_ESM.docx]

**Additional file 2: Table S1. Genes that are differentially expressed by stratified human keratinocytes from different anatomical sites after HPV16 transfection.**

| Upregulated  Genes   \| **FORESKIN** \| **TONSIL** \| **VAGINA** \| **GINGIVA** \| **CERVICAL** \| \| --- \| --- \| --- \| --- \| --- \| \| C1R \| CD8B \| AZGP1 \| BST2 \| AGER \| \| C1S \| GBP1 \| CXCL11 \| CCL20 \| APOBEC3F \| \| CD83 \| HLA-B \| DHX58 \| CCL27 \| APOBEC3G \| \| FOXF1 \| HLA-C \| GBP1 \| DEFB1 \| B2M \| \| HLA-F \| IFI27 \| HERC5 \| IL3 \| CCL22 \| \| IL1RL2 \| IFIT1 \| IFI6 \| IL31 \| CD40 \| \| NUP62 \| IFNA14 \| IFI27 \| IL11RA \| FAS \| \| UBE2V1 \| IRF5 \| IFIT2 \| IL17C \| GPX1 \| \|  \| IRF9 \| IFIT3 \| IL1RL2 \| HLA-E \| \|  \| MX1 \| IFNA14 \| IL22RA1 \| HLA-G \| \|  \| OAS2 \| IRF1 \| IL36B \| IFI30 \| \|  \| OASL \| MAPKAPK3 \| IL36G \| IRF7 \| \|  \| PSMB8 \| MX1 \| NECTIN1 \| OASL \| \|  \| SCRIB \| NCF2 \| OSMR \| PML \| \|  \| STAT1 \| OASL \| PROCR \| PNP \| \|  \| TAP2 \| STAT1 \| PTPN22 \| PRKCB \| \|  \| TAPBP \| TNFSF10 \|  \| PSMB8 \|   TRIM22 SP100  TIRAP  Down Regulated  Genes   \|  \|  \|  \|  \|  \| \| --- \| --- \| --- \| --- \| --- \| \| CAMK2G \| BCAP31 \| CD46 \| BPIFA1 \| CCL8 \| \| CCL20 \| CXCL13 \| IFNLR1 \| CD247 \| CLEC4C \| \| CORO1A \| IFNAR1 \| IKBKE \| DEFA1 \| IFNA10 \| \| CXCL1 \| NECTIN1 \| IRF3 \| ENAH \| IGLL1/IGLL5 \| \| CXCL16 \| OAS1 \| MAVS \| IFI30 \|  \| \| DDA1 \| TICAM1 \| OAS1 \| IL32 \|  \| \| DEFB4A/DEFB4B \| UNC93B1 \| PELI1 \| OSMR \|  \| \| FOXJ1 \|  \| TICAM1 \| P2RY14 \|  \| \| HLA-DMB \|  \| TLR5 \| PTAFR \|  \| \| IL1R1 \|  \|  \| SFTPD \|  \| \| IL36B \|  \|  \| TLR6 \|  \| \| IL36G \|  \|  \|  \|  \| \| KYNU \|  \|  \|  \|  \| \| PRKD2 \|  \|  \|  \|  \| \| RNF31 \|  \|  \|  \|  \| \| SIGIRR \|  \|  \|  \|  \| \| TAP2 \|  \|  \|  \|  \| \| TLR5 \|  \|  \|  \|  \| \| TNFSF14 \|  \|  \|  \|  \| \| TOLLIP  UNC93B1 \|  \|  \|  \|  \| |
| --- | --- | --- | --- | --- | --- | --- | --- | --- | --- | --- | --- | --- | --- | --- | --- | --- | --- | --- | --- | --- | --- | --- | --- | --- | --- | --- | --- | --- | --- | --- | --- | --- | --- | --- | --- | --- | --- | --- | --- | --- | --- | --- | --- | --- | --- | --- | --- | --- | --- | --- | --- | --- | --- | --- | --- | --- | --- | --- | --- | --- | --- | --- | --- | --- | --- | --- | --- | --- | --- | --- | --- | --- | --- | --- | --- | --- | --- | --- | --- | --- | --- | --- | --- | --- | --- | --- | --- | --- | --- | --- | --- | --- | --- | --- | --- | --- | --- | --- | --- | --- | --- | --- | --- | --- | --- | --- | --- | --- | --- | --- | --- | --- | --- | --- | --- | --- | --- | --- | --- | --- | --- | --- | --- | --- | --- | --- | --- | --- | --- | --- | --- | --- | --- | --- | --- | --- | --- | --- | --- | --- | --- | --- | --- | --- | --- | --- | --- | --- | --- | --- | --- | --- | --- | --- | --- | --- | --- | --- | --- | --- | --- | --- | --- | --- | --- | --- | --- | --- | --- | --- | --- | --- | --- | --- | --- | --- | --- | --- | --- | --- | --- | --- | --- | --- | --- | --- | --- | --- | --- | --- | --- | --- | --- | --- | --- |
